# Multilocus sequence analysis reveals different lineages of *Pseudomonas anguilliseptica* associated with disease in farmed lumpfish (*Cyclopterus lumpus* L.)

**DOI:** 10.1371/journal.pone.0259725

**Published:** 2021-11-22

**Authors:** Even Bysveen Mjølnerød, Hanne Katrine Nilsen, Snore Gulla, Andreas Riborg, Kirsten Liland Bottolfsen, Tom Wiklund, Debes Christiansen, Jesús Ángel López Romalde, Felix Scholz, Duncan John Colquhoun

**Affiliations:** 1 University of Bergen, Institute of Biological Science, Bergen, Norway; 2 Norwegian Veterinary Institute, Bergen, Norway; 3 Norwegian Veterinary Institute, Oslo, Norway; 4 Vaxxinova AS, Bergen, Norway; 5 Laboratory of Aquatic Pathobiology, Environmental and Marine Biology, Åbo Akademi University, Turku, Finland; 6 Food and Veterinary Agency, National Reference Laboratory for Fish Diseases, Tórshavn, Faroe Islands; 7 Department of Microbiology and Parasitology, CIBUS-Faculty of Biology & Institute CRETUS, Universidade de Santiago de Compostela, Santiago de Compostela, Spain; 8 FishVet Group Ireland, Galway, Ireland; Hokkaido University, JAPAN

## Abstract

The bacterium *Pseudomonas anguilliseptica* has in recent years emerged as a serious threat to production of lumpfish in Norway. Little is known about the population structure of this bacterium despite its association with disease in a wide range of different fish species throughout the world. The phylogenetic relationships between 53 isolates, primarily derived from diseased lumpfish, but including a number of reference strains from diverse geographical origins and fish species, were reconstructed by Multi-Locus Sequence Analysis (MLSA) using nine housekeeping genes (*rpoB*, *atpD*, *gyrB*, *rpoD*, *ileS*, *aroE*, *carA*, *glnS* and *recA*). MLSA revealed a high degree of relatedness between the studied isolates, altough the seven genotypes identified formed three main phylogenetic lineages. While four genotypes were identified amongst Norwegian lumpfish isolates, a single genotype dominated, irrespective of geographic origin. This suggests the existence of a dominant genotype associated with disease in production of lumpfish in Norwegian aquaculture. Elucidation of the population structure of the bacterium has provided valuable information for potential future vaccine development.

## Introduction

Introduction and widespread use of cleaner fish, such as lumpfish (*Cyclopterus lumpus*), for control of sea lice infestations (*Lepeoptheirus salmonis* and *Caligus elongatus*) in Atlantic salmon (*Salmo salar*) aquaculture in Norway presents novel virologic, parasitic and bacteriological issues. Since the start of production of farmed lumpfish, the bacteria most commonly associated with disease in this fish species have been *Vibrio anguillarum* [1],‘atypical’ *Aeromonas salmonicida* [2], *Moritella viscosa*, *Tenacibaculum* spp., and *Pasteurella* sp. [3, 4]. Following its first isolation in 2013, *Pseudomonas anguilliseptica* has however emerged as an additional and serious threat to lumpfish production in Norway, and the number of outbreaks has since increased annually [5]. *P*. *anguilliseptica* associated mortalities have also been reported in lumpfish farmed in Scotland [6], Ireland, and the Faroe Islands (Scholz; Christiansen; unpublished data).

*P*. *anguilliseptica* is recognized as an opportunistic pathogen primarily affecting fish in marine and brackish environments. It was originally described in 1971 as the causative agent of “red spot disease” (sekiten-byo) in pond cultured Japanese eel (*Anguilla japonica*). Isolated in 1981 from European eel (*Anguilla anguilla*) in Scotland [7], the bacterium became a frequently occurring pathogen associated with eel farming throughout Europe. Initially, *P*. *anguilliseptica* was considered to be exclusively associated with disease in eel culture [8]. However, the bacterium has since been recognized to display little or no host specificity as it has been shown to be pathogenic in a range of cultured and wild fish species in different geographic areas (Table 1). With the exception of a small number of isolates from farmed wolffish (*Anarhichadidae*) and a single isolation from seawater farmed rainbow trout (*Oncorhynchus mykiss*) in 2018 [9], *P*. *anguilliseptica* infection appears to be particularly associated with disease in lumpfish in Norway.

**Table 1:** Species of wild caught and cultured fish shown to develop disease due to infections with *P*. *anguilliseptica*.

Species		Geographic origin	Reference
Japanese eel	*Anguilla japonica*	Japan	[10]
European eel	*Anguilla anguilla*	Scotland	[7]
Ayu	*Plecolossus altivelis*	Japan	[11]
Atlantic salmon	*Salmo salar*	Finland	[12]
Sea trout	*Salmo trutta*	Finland	[12]
Whitefish	*Coregonus* sp.	Finland	[12]
Rainbow trout	*Onchorhynchus mykiss*	Finland	[12]
Baltic herring	*Clupea harengus*	Baltic sea	[13]
Sea bass	*Dicentrarchus labrax*	France	[14]
Turbot	*Scophthalmus maximus*	Spain	[15]
Gilthead seabream	*Sparus aurata*	France, Spain	[14, 16]
Black spot seabream	*Pegallus bogoraveo*	Spain	[17]
Cod	*Gadus morhua*	UK/Canada, Scotland	[18, 19]
Striped beakperch	*Oplegnathus fasciatus*	Korea	[20]
Lumpfish	*Cyclopterus lumpus*	Norway	[3]

Little is known of the population structure of this bacterium despite its association with disease in numerous fish species over a wide geographic range. While phenotypic studies [12, 13, 21], identified few differences, Random Amplification of Polymorphic DNA (RAPD) fingerprinting separated the population into two genetic clades, with one clade almost exclusively represented by eel isolates.

A subsequent serological study [22] identified two serologically distinct groups, termed serotypes O1 and O2, consistent with the two RAPD groups identified previously. While serotype O1 were isolated from a diverse range of fish species, serotype O2 isolates were almost exclusively associated with eels. The concordance observed between the genetic and serologic characteristics culminated in the proposal of two separate clonal lineages for the bacterium [23].

Given the genetic and serological diversity demonstrated within the species and existence of both species-specific variants and broad host range variants, the aim of the present study was to elucidate the phylogenetic relationships amongst Norwegian isolates of *P*. *anguilliseptica* isolated from farmed lumpfish. A multilocus sequence analysis (MLSA) scheme comprising nine housekeeping (HK) genes was therefore designed to phylogenetically reconstruct the population structure of a variety of host derived, geographically disparate and historically distinct isolates of *P*. *anguilliseptica*. The knowledge generated may shed light on infection dynamics, allow development of specific diagnostics and identify strains relevant for future vaccine development.

## Materials and methods

### Confirmation of isolate identity

A total of 53 isolates were included in the analysis. These were sampled from a range of different spatiotemporal origins and fish species (Table 2). All isolates originated from clinically infected fish and were cultivated on blood agar w/2% NaCl at 15°C for 48 hours. Matrix Assisted Laser Desorption/Ionization Time-of-Flight Mass Spectrometry (MALDI-TOF) was performed on all isolates, while 16S rRNA gene sequencing and/or phenotypic characterization was used in some cases to positively confirm species identity prior to the MLSA.

**Table 2:** Isolates of *Pseudomonas anguilliseptica* included in this study.

Isolate number	Year of isolation	Strain	Serotype	Fish species	Origin of isolate	Source
NCIMB 1949^T^	1998	NCIMB 1949	O2	European eel	Japan	NCIMB
NVIO 11299	2018			Lumpfish	Ireland	FVGI
NVIO 11300	2018			Lumpfish	Ireland	FVGI
NVIO 11301	2018			Lumpfish	Ireland	FVGI
NVIO 11302	2018			Lumpfish	Ireland	FVGI
NVIO 11303	2018			Lumpfish	Ireland	FVGI
NVIO 9942	2015			Lumpfish	Faroe Islands	FFVA
NVIO 10973	2017			Lumpfish	Faroe Islands	FFVA
NVIO 11158	2017			Lumpfish	Faroe Islands	FFVA
NVIO 11159	2017			Lumpfish	Faroe Islands	FFVA
NVIO 11160	2017			Lumpfish	Faroe Islands	FFVA
NVIO 11161	2017			Lumpfish	Faroe Islands	FFVA
NVIO 11162	2017			Lumpfish	Faroe Islands	FFVA
NVIO 8180	2011			Lumpfish	Norway	NVI
NVIO 8227	2012			Lumpfish	Norway	NVI
NVIO 9976	2015			Lumpfish	Norway	NVI
NVIO 10039	2015			Lumpfish	Norway	NVI
NVIO 10341	2016			Lumpfish	Norway	NVI
NVIO 10449	2016			Lumpfish	Norway	NVI
NVIO 10550	2016			Lumpfish	Norway	NVI
NVIO 10726	2016			Lumpfish	Norway	NVI
NVIB 50–927	2015			Lumpfish	Norway	NVI
NVIB 50–1353	2016			Lumpfish	Norway	NVI
NVIB 50–1579	2016			Lumpfish	Norway	NVI
NVIB 50–1705	2016			Lumpfish	Norway	NVI
NVIB 50–1763	2017			Lumpfish	Norway	NVI
NVIB 50–1825	2017			Lumpfish	Norway	NVI
NVIB 50–1846	2017			Lumpfish	Norway	NVI
NVIB 50–1895	2017			Lumpfish	Norway	NVI
NVIB 50–1910	2017			Lumpfish	Norway	NVI
NVIB 50–1914	2017			Lumpfish	Norway	NVI
NVIB 50–1952	2017			Lumpfish	Norway	NVI
NVIB 50–2015	2017			Lumpfish	Norway	NVI
NVIB 50–2095	2017			Lumpfish	Norway	NVI
NVIB 50–2040	2017			Lumpfish	Norway	NVI
NVIO 11313	2018			Lumpfish	Norway	NVI
NVIO 11370	2019			Lumpfish	Norway	NVI
NVIO 8905	2013			Wolffish (*Anarhichas lupus*)	Norway	NVI
NVIB 50–2084	2017			Ballan wrasse (Labrus bergylta)	Norway	NVI
NVIB 50–2255	1992	STR-6		Baltic herring	Baltic sea	ÅAU
NVIB 50–2260	1992	STR2-1		Baltic herring	Baltic sea	ÅAU
NVIB 50–2256	1991	P57B/91		Rainbow trout	Finland	ÅAU
NVIB 50–2257	1993	0506-F05		Brown trout (*Salmo trutta*)	Finland	ÅAU
NVIB 50–2258	2000	P19/00	O1	Atlantic salmon	Finland	ÅAU
NVIB 50–2259	2008	P33-6/08		Whitefish	Finland	ÅAU
NVIB 50–2262	2015	P17-3/15		Whitefish	Finland	ÅAU
NVIB 50–2261	2009	P30-5/09		Rainbow trout	Finland	ÅAU
NVIO 11214	2018	AZ/210-1	O1	Turbot	Spain	CIBUS
NVIO 11215	2018	AZ/210-2	O1	Turbot	Spain	CIBUS
NVIO 11216	2018	AZ/211-1	O1	Turbot	Spain	CIBUS
NVIO 11217	2018	AZ/211-2	O1	Turbot	Spain	CIBUS
NVIO 11219	2018	TW47/L1	O1	Gilthead Sea Bream	Spain	CIBUS
NVIO 11220	2018	TW75/L3		Gilthead Sea Bream	Spain	CIBUS

Source annotation: NCIMB (National Collection of Industrial Food and Marine Bacteria), FVGI (Fish Vet Group Ireland), FFVA (Faraoese Food and Veterinary Agency), NVI-B/O (Norwegian Veterinary Institute, Bergen/Oslo), ÅAU (Åbo Akademi University), CIBUS (Centro de Investigaciones Biológicas, Universidade de Santiago de Compostela). ^*T*^ Type strain NCIMB 1949.

**MALDI-TOF.** MALDI-TOF analysis was performed on a Biotyper Microflex LT (Bruker Daltonics, Bremen, Germany). In-house main spectra (MSP) were generated for *P*. *anguilliseptica* isolate NVIB 50–2084 and type strain NCIMB 1949. For MSP generation, proteins from each reference strain were extracted according to the manufacturer’s protocol (S1 Protocol).

Following incorporation of constructed MSPs into the reference library, single colonies of putative *P*. *anguilliseptica* were subjected to MALDI-TOF analysis by the Direct Transfer Method according to the manufacturer’s instructions. Briefly, single colonies were smeared with a toothpick as a thin film onto two successive spots on a MALDI target plate. After drying at room temperature, the spots were overlaid with HCCA matrix solution (saturated α-cyano-4-hyoxycinnamic acid in 50% acetonitrile 2,5% trifluoracetic acid) and air-dried. Identification was then performed using the standard Biotyper Database supplemented with the in house generated MSPs. Similarity scores ≥2.0 in relation to any particular MSP were considered to represent good identification to the species/genomovar level.

**Phenotypic characterization.** Phenotypic characterization was conducted on a representative selection of six isolates derived from various fish host species and geographic-/temporal origins (NVIO 10973, NVIO 8905, NVIO 11214, NVIB 50–2255, NVIB 50–1910 and NVIO 9976; Table 2). The characteristics examined were: colony morphology, gram staining, motility, anaerobic respiration and hemolysis on blood agar. Biochemical properties such as oxidative/fermentative (O/F) production of acid from glucose, as well as the ability to produce decarboxylases from the amino acids arginine, lysine and ornithine (ALO), were also tested. Production of oxidase was tested using the DrySlide Oxidase (BD, Franklin Lakes, USA).

### DNA extraction, PCR and sequencing

Template DNA was isolated using the High Pure PCR Template Preparation Kit (Roche Applied Science, Penzberg, Germany) in accordance with the manufacturer’s protocol. Concentration and purity of nucleic acid in the final eluate was assessed with the NanoDrop 2000 Spectrophotometer (ThermoFisher Scientific, Waltham, USA). All samples were diluted to 2 ng/μl before being stored at -20°C.

The following housekeeping (HK) genes were included in the MLSA assay: *rpoB* (RNA polymerase beta subunit), *atpD* (ATP synthase F1 beta subunit), *gyrB* (DNA gyrase beta subunit), *rpoD* (RNA polymerase, sigma factor), *ileS* (isoleucyl-tRNA synthetase), *aroE* (shikhimate dehydrogenase), *carA* (carbamoyl-phosphate synthase small chain), *glnS* (glutaminyl-tRNA synthetase), and *recA* (recombinase A). PCR primers were designed for each of the nine housekeeping genes based on homologous sequences from *Pseudomonas aeruginosa*, *P*. *orzihabitans* and *P*. *anguilliseptica* Type strain DSM 12111^T^, retrieved from public databases (S1 Table). Alignment and primer design was performed in Geneious (Biomatters, Auckland, New Zealand). Primers are listed in Table 3. Forward and reverse M13 primer sequences (Thermo Fisher Scientific) were added to each primer set for downstream sequencing of PCR amplicons. M13 Forward: 5´d[GTAAAACGACGGCCAG]3´, M13 Reverse: 5´d[CAGGAAACAGCTATGAC]3´.

**Table 3:** Primers (without M13) for each of the nine housekeeping genes included in the analysis.

Primer	Sequence (5´- 3´)	Gene product	Size of target sequence (bp)	PCR product post trim (bp)	Annealing temp (°C)	Reference
aroE-42 F	CAAGTCGCCGCTGATTCATC	Shikimate dehydrogenase	653	504	56°C	This study
aroE-761 R	GTTCGACCAGCATGCCCAG					
atpD-114 F	ACCCTGGAAGTTCAGCAGC	ATP synthase F1, beta subunit	808	645	56°C	This study
atpD-965 R	TACAACGGTGGCGTCCAAG					
carA-143 F	CCGATCCTTCCTATGCCCAG	Carbamoyl-phosphate synthase	762	525	56°C	This study
carA-956 R	GTTCTGGCTGGTGATCATCAC					
glnS-238 F	CGCCAAGGAAGACCAGGAG	Glutaminyl-tRNA synthetase	587	528	56°C	[24]
glnS-863 R	CTTGCGCTTGCTGGTAATCG					
ileS-43 F	TTTCCGATGAAGGCCGGC	Isoleucyl-tRNA synthetase	708	645	56°C	[24]
ileS-788 R	GGTAAACTCCGGGTGAACGT					
rpoB-3,307 F	TGTGGTCTCGGTGATCATGC	RNA polymerase, beta subunit	529	504	56°C	[24]
rpoB-3,878 R	GAACTGCGCCTTACCACCC					
rpoD-294 F	GACCCAGTGCGCATGTACAT	RNA polymerase sigma factor	766	732	56°C	This study
rpoD-1,204 R	ATGCGACGGTTGATGTCCTT					
recA-136 F	CTCGCTTGGTCTGGACATCG	Recombinase A	740	636	56°C	This study
recA-959 R	CCTTGCCTTGGCCGATCTT					
gyrB-329 F	ACAGCTACAAGGTTTCCGGC	DNA gyrase, beta subunit B	703	645	56°C	This study
gyrB-1,089 R	CTTGCCCATTTCCTGCTCGA					

Amplification of HK genes by PCR was based on a standard reaction mixture containing (per reaction) 4 μl 5xGreen GoTaq Flexi buffer, 1.5 μl MgCl_2_ Solution (25 mM), 0.4 μl dNTP (10 mM), 1 μl of forward and reverse primers (10 μM), 0.1 μl Go Taq G2 Flex DNA polymerase, 8 μl nuclease-free water, and 4 μl DNA template, amounting to a total reaction volume of 20 μl. All reagents were supplied by Promega (Madison, USA).

PCR was performed in an Agilent SureCycler 8800 Thermal Cycler (Santa Clara, USA) and involved 95°C for 3 min, 30 cycles of denaturation (95°C for 1 min), annealing (56°C for 1 min) and elongation (72°C for 1 min), followed by 72°C for 5 min and cooling to 4°C indefinitely. Amplification of PCR-products of the desired size was confirmed by gel electrophoresis using the GelPilot® 100bp Plus Ladder (QIAGEN, Venlo, Netherlands) as reference. PCR products were purified with AMPureXP® (Beckman Coulter, Pasadena, USA) using the Biomek4000 pipetting robot (Beckman Coulter, Pasadena, USA).

Cycle sequencing of purified PCR products was based on a reaction mixture containing (per reaction) 0.5 μl BigDye v3.1, 2.0 μl 5X Sequencing Buffer, 2.0 μl M13 primer (2.5 μM), 2.0 μl purified PCR product and 3.5 μl MilliQ-water, and involved 96°C for 1 min, 25 cycles of 96°C for 10 seconds, 50°C for 5 seconds and 60°C for 4 min, followed by cooling to 4°C indefinitely. This was performed using an Applied Biosystems® Veriti® 96-Well Thermo Cycler (ThermoFisher Scientific, Waltham, USA). Sequence products were purified using the BigDye® XTerminator Purification Kit (ThermoFisher Scientific, Waltham, USA) in accordance with its protocol. Capillary electrophoresis of purified sequence products was performed using the 3500XL Genetic Analyzer (ThermoFisher Scientific, Waltham, USA) according to the manufactures protocol.

### Data analysis

Forward and reverse strands for each amplicon were sequenced for all isolates and imported to Geneious 11.1.5 (Biomatters, Auckland, New Zealand) for contig assembly and further processing. Contigs were manually checked and edited and re-sequencing performed when necessary. Contigs were then trimmed at the 5´and 3´-ends to exclude primer sequences and areas of poorer quality towards the amplicon ends. All sequences were trimmed to preserve the reading frame. Final processed sequences were imported and concatenated in Microsoft Excel. Concatenated sequences were then re-imported to Geneious to construct the final alignment. Complete record of partial gene sequences for all isolates was submitted to GenBank (accession nos. MW684870—MW685347).

### Multi-Locus sequence analysis

Based on the concatenated alignment, a maximum likelihood phylogenetic reconstruction was run in MEGA X using the TN93 substitution model [25] (identified as optimal by the model test module). Number of bootstraps was set to 1000, while all other inputs were set to default. The phylogenies were similarly reconstructed for individual genes and for a concatenated amino acid alignment (LG substitution model [26] used for the latter).

## Results

### Confirmation of isolate identity

MALDI-TOF analysis of samples submitted for the study positively identified all 53 isolates as *P*. *anguilliseptica* with similarity scores ≥2.0. 16S rRNA gene sequencing of 39 isolates also demonstrated concordance with MALDI-TOF results.

With the exception of production of arginine dihydrolase (isolate NVIO 8905 negative), the studied isolates were phenotypically similar. The results of phenotypical testing are listed in Table 4.

**Table 4:** Phenotypic characteristics of six *P*. *anguilliseptica* isolates (isolate number, geographic origin and time of isolation).

	NVIO 10973	NVIO 8905	NVIO 11214	NVIB 50–2255	NVIB 50–1910	NVIO 9976
	Lumpfish, Faroe Islands	Wolffish, Norway	Turbot, Spain	Baltic herring, Baltic Sea	Lumpfish, Norway	Lumpfish, Norway
	2017	2013	2018	1992	2017	2015
Test						
Gram	−	−	−	−	−	−
Motility	+	+	+	+	+	+
Cytochrome oxidase	+	+	+	+	+	+
O/F	−	−	−	−	−	−
Anaerobe respiration	−	−	−	−	−	−
Arginine dihydrolase	+	−	+	+	+	+
Lysine decarboxylase	−	−	−	−	−	−
Ornithine decarboxylase	−	−	−	−	−	−
Vibriostatic sensitivity	−	−	−	−	−	−
Haemolysis on blood agar	−	−	−	−	−	−

For dihydrolase/decarboxylase testing + denotes a strong purple reaction while–denotes a weak or lack of colour change.

### Multi-Locus sequence analysis

**Fig 1:**
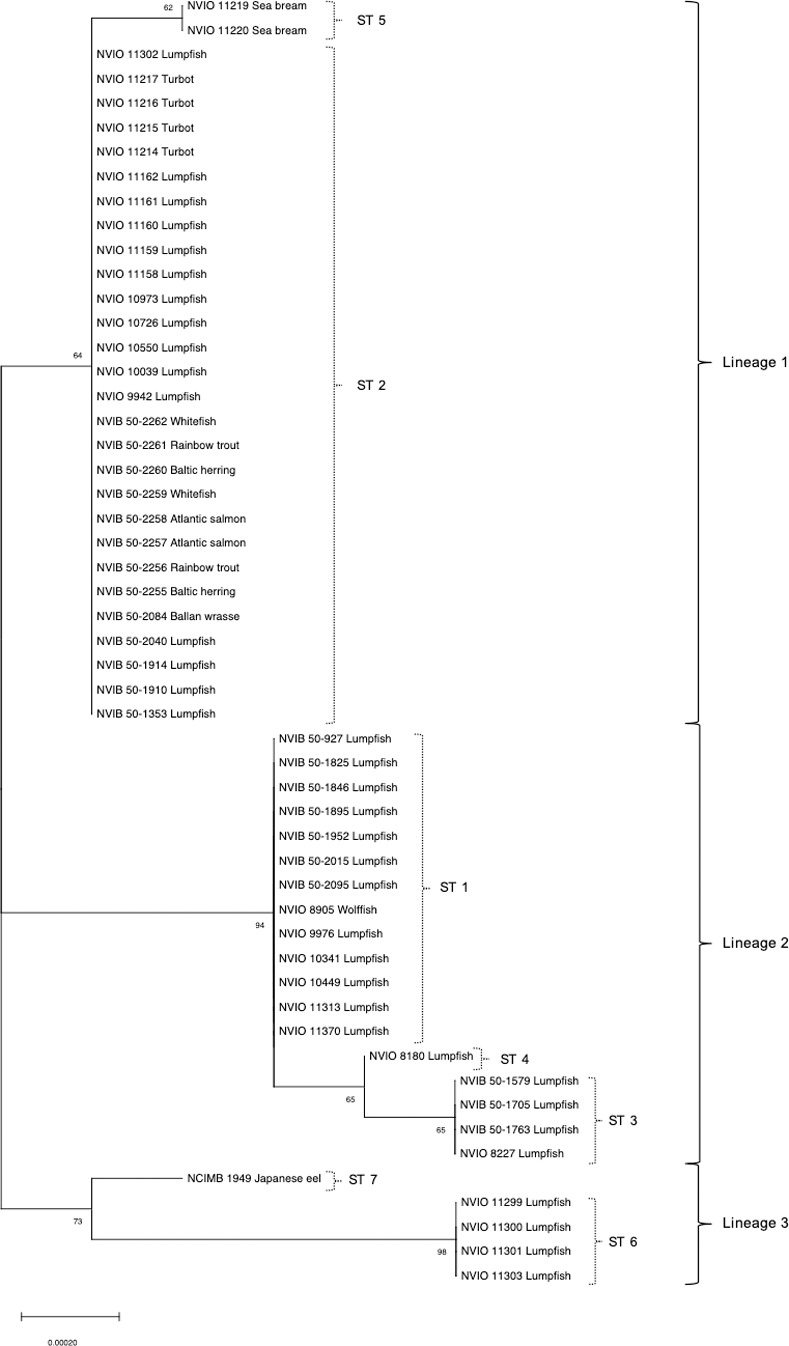
Phylogenetic reconstruction (MLSA) of 53 isolates of *P*. *anguilliseptica* based on the concatenated *aroE*, *recA*, *rpoB*, *ileS*, *rpoD*, *glnS*, *atpD*, *gyrB* and *carA* gene sequences. Bootstrap support values shown next to the branches. The length of the scale bar corresponds to 0.00020 substitutions per site. Stapled brackets refer to STs identified. Unstapled brackets refer to the major lineages formed following the phylogenetic reconstruction. Annotation: Norwegian Veterinary Institute Bergen/Oslo (NVIB/NVIO), isolate number, fish species.

**Fig 2:**
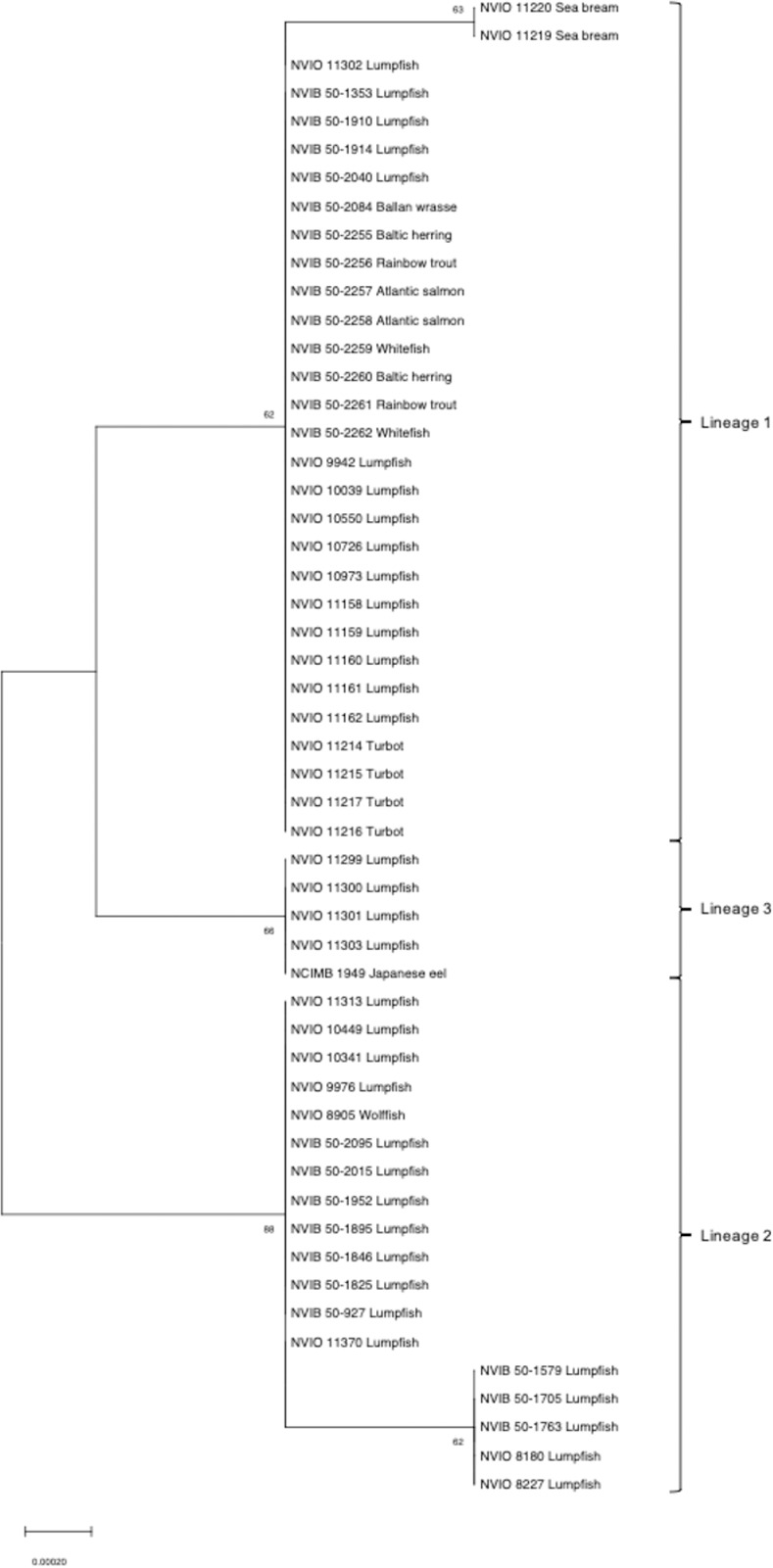
Phylogenetic reconstruction of 53 isolates of *P*. *anguilliseptica* based on concatenated *aroE*, *recA*, *rpoB*, *ileS*, *rpoD*, *glnS*, *atpD*, *gyrB* and *carA* amino acid sequences. Bootstrap support values are shown next to the branches. The length of the scale bar corresponds to 0.00020 substitutions per site. Brackets refer to the major lineages formed following the phylogenetic reconstruction. Annotation: Norwegian Veterinary Institute Bergen/Oslo (NVIB/NVIO), isolate number, fish species.

The concatenated alignment of nine housekeeping genes (5,364bp) displayed average sequence identities of 99.9% between all isolates. Phylogenies inferred from MLSA based on nucleotide sequences revealed seven genotypes distributed amongst three major lineages (Fig 1). Inspection of the tree shows that lumpfish-isolates are dispersed thoughout depending on country of origin (i.e. Norwgian isolates in lineages 1 and 2, Faroese in lineage 1 and Irsish in lineages 1 and 3), while isolates from other fish species (irrespective of geography) belong almost exclusively to lineage 1. The type strain NCIMB 1949 from Japanese eel in Japan constitutes one exception to this, as it forms a singleton genotype within lineage 3. Phylogenetic reconstruction based on concatenated amino acid sequences displayed compatible topologies (Fig 2). Maximum likelihood trees generated from single gene alignments displaying genetic heterogeneity (*rpoD*, *rpoB*, *carA*, and *atpD*) are attached as (S1–S4 Figs). For *aroE*, *glnS*, *ileS*, *recA* and *gyrB*, complete genetic homogeneity was observed across all 53 isolates.

## Discussion

The present work represents the most comprehensive phylogenetic study to date of the fish pathogenic bacterium *P*. *anguilliseptica* originating from a range of different geographic localities, fish species and time of isolation.

MLSA revealed a high degree of genetic similarity irrespective of geographic origin, fish host species or time of isolation. The significant degree of genetic conservation identified amongst isolates retrieved from a wide geographic area over a period of 21 years suggest ‘purifying’ selection pressure towards a restricted fish parasitic/pathogenic niche. Evolution of virulent host associated lineages from ancestral environmental strains has previously been described from *Francisella tularensis* [27]. As in previous studies relating to *P*. *anguilliseptica*, all isolates studied here originated from clinically infected fish. No environmental isolates were available. Had such isolates been available they may have revealed a greater intraspecific diversity. While the possibility that fish pathogenic *P*. *anguilliseptica* represent expansion of a fish pathogenic clone against a background of environmental, possibly non-virulent strains cannot be ruled out, there is, however, no literature published to date suggesting the existence of environmental strains of this bacterium.

A degree of phylogenetic heterogeneity was, however identified. The phylogenetic patterns identified appear to be more related to geographic origin than chronology or host fish species. The Norwegian isolates, originating with the exception of two isolates from wolffish and ballan wrssse, from farmed lumpfish, were distributed between two of the three major lineages (lineage 1 and 2, Fig 1). Lineage 1 is the most numerous of the three, with isolates originating from most geographic localities and fish species. Approximately a third of all Norwegian isolates are situated within this lineage, while the rest are situated in the exclusively Norwegian lineage 2. Although the Norwegian isolates were distributed amongst two phylogenetic lineages, no distinct differences were registered between these lineages in terms of domestic geography or time of isolation. MLSA based on concatenated amino acid sequences displayed a pattern consistent with nucleotide sequence analysis in the formation of three major lineages.

Following identification of the various lineages by MLSA, MALDI-TOF MSP’s were generated from NVIB 50–2084 and NCIMB 1949, representing MLSA lineages 1 and 3 respectively (Fig 1). Subsequent MALDI-TOF analysis of the entire study collection utilizing these MSP’s revealed that MALDI-TOF may be capable, with further refinement, of distinguishing between the different lineages observed, and may therefore prove a valuable future diagnostic tool for discrimination of these lineages.

The results of phenotypic characterization were in compliance with previously published descriptions [12, 13, 21] with the exception of a single isolate which was negative for production of arginine dihydrolase (Table 4). A complementary analysis of all isolates included in the present study showed three isolates (NVIO 8827, NVIO 8905, and NVIB 50–1579), all situated within lineage 2, negative for arginine dihydrolase production. This appears to be a variable trait only within this lineage. The existence of phenotypic variation beyond the phylogenetic resolution achieved through MLSA can thus not be ruled out.

Previous studies have suggested the existence of antigenic variation within *P*. *anguilliseptica*. Seven of the isolates studied in López-Romalde et al. [15, 22] were used in the present study (see Table 1). All included isolates representing serotype O1 were situated within the same phylogenetic lineage (Fig 1) and it is reasonable to assume that all isolates within this lineage probably belong to serotype O1.

Notably, all but one of the Irish lumpfish isolates included in this study clustered closely with the type strain NCIMB 1949, isolated from Japanese eel in Japan, within lineage 3 (Fig 1). The serological differences identified by López Romalde et al. [22] between representatives of lineage 1 (O1) and NCIMB 1949 (O2) suggest that these Irish isolates might potentially also belong to serotype O2. Future antigenic characterization would be required to determine whether this is the case. Irish isolate NVIO 11302 deviates, however, from this pattern and is situated within the geographically spread lineage 1 (Fig 1). This situation could either be due to the existence of several genotypes associated with disease in Irish lumpfish or possibly indicate spread of infection via import of Norwegian lumpfish-roe to Ireland.

Of the Norwegian isolates, 8 of 26 were situated within lineage 1, likely representing serotype O1. The remaining, and thereby the majority of Norwegian isolates, were situated in lineage 2, which shows comparable phylogenetic separation from lineage 1 as are at least as phylogenetically distant, if not slightly more distant to lineage 1 than NCIMB 1949 (serotype O2) (Fig 1). This raises the question as to whether lineage 2 may represent a previously yet undescribed serotype. The fact that all Norwegian isolates are distributed between two of the major phylogenetic lineages (1 and 2), possibly representing different serotypes, complicates identification of a suitable candidate strain for vaccine development. Characterization of the serological properties of Norwegian isolates will therefore be necessary with respect to the potential development of a mono- or multi-genotype vaccine for future use in lumpfish.

Interestingly, isolates of identical genotype were identified from Norwegian lumpfish and diseased Atlantic salmon in Finland (Fig 1). The identical genotype and the pathogenic nature of the bacterium [12] suggests that *P*. *anguilliseptica* might potentially pose a threat to cohabiting salmonids in commercial net pens in Norway. However, considering the ever-increasing number of outbreaks among lumpfish and the lack of reported infections in cohabiting salmon, this indicates that Norwegian salmon are not highly susceptible to *P*. *anguilliseptica* infection. While future whole genome sequencing studies may reveal sub-MLSA genotype differences related to host specificity, establishment of a challenge model to test salmon susceptibility to the Norwegian lumpfish strain should be considered to evaluate the potential risk of cross-species transmission.

## Conclusion

The nine-locus MLSA employed in this study identified a considerable degree of genetic similarity among isolates of *P*. *anguilliseptica*, separating the studied isolates amongst seven closely related genotypes. Several genotypes were identified amongst Norwegian lumpfish isolates, although the majority belonged a single overarching lineage. Antigenic study of representative isolates from each genotype should be evaluated as a basis for development of vaccines against this important lumpfish pathogen.

## Supporting information

S1 Protocol MALDI-TOF Main Spectra Profile (MSP) generation for *P*. *anguilliseptica*. (DOCX)

S1 Table Locus accession numbers (NCBI) for *P*. *aeruginosa*, *P*. *orysihabitans* and *P*. *anguilliseptica* for alignment and PCR primer design. (DOCX)

S1 Fig Maximum likelihood tree based on *rpoD* sequence (766 bp). (DOCX)

S2 Fig Maximum likelihood tree based on *rpoB* sequence (507 bp). (DOCX)

S3 Fig Maximum likelihood tree based on *carA* sequence (526 bp). (DOCX)

S4 Fig Maximum likelihood tree based on *atpD* sequence (645 bp). (DOCX)

## References

[1] Marcos-López M, Donald K, Stagg H, McCarthy Ú. Clinical *Vibrio anguillarum* infection in *Cyclopterus lumpus* in Scotland. Vet Rec. 2013;173: 319.2–319. doi: 10.1136/vr.101763 24008994

[2] Rønneseth A, Haugland GT, Colquhoun DJ, Wergeland HI, Brudal E, Wergeland HI, et al. Protection and antibody reactivity following vaccination of lumpfish (Cyclopterus lumpus L.) against atypical Aeromonas salmonicida. Fish Shellfish Immunol. 2017/03/28. 2017;64: 383–391. doi: 10.1016/j.fsi.2017.03.040 28344167

[3] Alarcón M, Gulla S, Røsæg M V, Rønneseth A, Wergeland H, Poppe TT, et al. Pasteurellosis in lumpsucker (Cyclopterus lumpus), farmed in Norway. J Fish Dis. 2016. doi: 10.1111/jfd.12366 25828053

[4] Ellul RM, Walde C, Haugland GT, Wergeland H, Rønneseth A. Pathogenicity of Pasteurella sp. in lumpsuckers (Cyclopterus lumpus L.). J Fish Dis. 2018. doi: 10.1111/jfd.12905 30311669

[5] Walde C, Gulla S, Hansen H, Bysveen Mjølnerød E, Bornø G. Helsesituasjonen hos rensefisk. In: BBornø Hjeltnes, GHaukaas, AWalde, BBJ C, editor. Fiskehelserapporten 2018. https://www.vetinst.no/rapporter-og-publikasjoner/rapporter/2019/fiskehelserapporten-2018; 2019. pp. 119–125.

[6] Treasurer JW, Birkbeck TH. Pseudomonas anguilliseptica associated with mortalities in lumpfish (Cyclopterus lumpus L.) reared in Scotland. Bull Eur Assoc FISH Pathol. 2018;38: 222–224.

[7] Nakai T, Muroga K. Pseudomonas anguilliseptica isolated from European eels (Anguilla anguilla) in Scotland. Fish Pathol. 1982;17: 147–150.

[8] Wiklund T. Pseudomonas anguilliseptica infection as a threat to wild and farmed fish in the Baltic Sea. Microbiol Aust. 2016;37: 135–136. doi: 10.3354/dao021151

[9] Colquhoun DJ, Nilsen H. Andre bakterieinfeksjoner hos laksefisk. In: Sommerset I, Walde C, Bang Jensen B, Bornø G, Haukaas A, Brun E, editors. Fiskehelserapporten 2019. https://www.vetinst.no/rapporter-og-publikasjoner/rapporter/2020/fiskehelserapporten-2019: Norwegian Veterinary Institute; 2019. p. 87.

[10] Wakabayashi H, Egusa S. Characteristics of a Pseudomonas sp. from an epizootic of pond-cultured eels (Anguilla japonica). Bull Japanese Soc Sci Fish. 1972;38: 577–587.

[11] Nakai T, Hanada H, Muroga K. First records of Pseudomonas anguilliseptica infection in cultured ayu, Plecoglossus altivelis. Fish Pathol. 1985;20: 481–484. doi: 10.3147/jsfp.20.481

[12] Wiklund T, Bylund G. Pseudomonas anguilliseptica as a pathogen of salmonid fish in Finland. Dis Aquat Organ. 1990;8: 13–19. doi: 10.3354/dao008013

[13] Lönnström L, Wiklund T, Bylund G. Pseudomonas anguilliseptica isolated from Baltic herring Clupea harengus membras with eye lesions. Dis Aquat Organ. 1994;18: 143–147. doi: 10.3354/dao018143

[14] Berthe FCJ, Michel C, Bernardet JF. Identification of *Pseudomonas anguilliseptica* isolated from several fish species in France. Dis Aquat Organ. 1995;21: 151–155. doi: 10.3354/dao021151

[15] López-Romalde S, Magariños B, Núñez S, Toranzo AE, Romalde JL. Phenotypic and genetic characterization of Pseudomonas anguilliseptica strains isolated from fish. J Aquat Anim Health. 2003;15: 39–47.

[16] Doménech A, Fernández-Garayzábal JF, García JA, Cutuli MT, Blanco M, Gibello A, et al. Association of Pseudomonas anguilliseptica infection with “winter disease” in sea bream, Sparus aurata L. J Fish Dis. 1999. doi: 10.1046/j.1365-2761.1999.00124.x

[17] López-Romalde S, Nuñez S, Toranzo AE, Romalde JL. Black spot seabream (Pagellus bogaraveo), a new susceptible host for Pseudomonas anguilliseptica. Bull Eur Assoc FISH Pathol. 2003;23: 258–264.

[18] Balboa S, Ferguson HW, Romalde JL. Phenotypic, serological and genetic characterization of Pseudomonas anguilliseptica strains isolated from cod, Gadus morhua L., in northern Europe. J Fish Dis. 2007. doi: 10.1111/j.1365-2761.2007.00849.x 17958609

[19] Ferguson HW, Collins RO, Moore M, Coles M, MacPhee DD. Pseudomonas anguilliseptica infection in farmed cod, Gadus morhua L. J Fish Dis. 2004/03/31. 2004;27: 249–253. doi: 10.1111/j.1365-2761.2004.00537.x 15049894

[20] Kim S-R, Park M-A, Kitamura S-I, Jung S-J, Oh M-J, Park M-A, et al. Recovery of Pseudomonas anguilliseptica from Diseased Striped Beakperch (Oplegnathus fasciatus) in Korea. Fish Aquat Sci. 2010;13: 190–194. doi: 10.5657/fas.2010.13.2.190

[21] Doménech A, Fernández-Garayzábal JF, Lawson P, García JA, Cutuli MT, Blanco M, et al. Winter disease outbreak in sea-bream (Sparus aurata) associated with Pseudomonas anguilliseptica infection. Aquaculture. 1997;156: 317–326. doi: 10.1016/s0044-8486(97)00069-0

[22] López-Romalde S, Magariños B, Ravelo C, Toranzo AE, Romalde JL, Lopez-Romalde S, et al. Existence of two O-serotypes in the fish pathogen Pseudomonas anguilliseptica. Vet Microbiol. 2003/06/28. 2003;94: 325–333. doi: 10.1016/s0378-1135(03)00124-x 12829386

[23] Romalde JL. Evidences of two clonal lineages within the emerging fish pathogen Pseudomonas anguilliseptica by serological and genetic techniques. AFS/FHS Newslett. 2003;31: 8–9.

[24] Andreani NA, Martino ME, Fasolato L, Carraro L, Montemurro F, Mioni R, et al. Reprint of “Tracking the blue: a MLST approach to characterise the Pseudomonas fluorescens group.” Food Microbiol. 2014/12/08. 2015;45: 148–158. doi: 10.1016/j.fm.2014.11.011 25481072

[25] Tamura K, Nei M. Estimation of the number of nucleotide substitutions in the control region of mitochondrial DNA in humans and chimpanzees. Mol Biol Evol. 1993/05/01. 1993;10: 512–526. doi: 10.1093/oxfordjournals.molbev.a040023 8336541

[26] Le SQ, Gascuel O. An improved general amino acid replacement matrix. Mol Biol Evol. 2008/03/28. 2008;25: 1307–1320. doi: 10.1093/molbev/msn067 18367465

[27] Larsson P, Elfsmark D, Svensson K, Wikstrom P, Forsman M, Brettin T, et al. Molecular evolutionary consequences of niche restriction in Francisella tularensis, a facultative intracellular pathogen. PLoS Pathog. 2009/06/13. 2009;5: e1000472. doi: 10.1371/journal.ppat.1000472 19521508 PMC2688086

