# Supplementary material for: Multilocus sequence analysis reveals different lineages of Pseudomonas anguilliseptica associated with disease in farmed lumpfish (Cyclopterus lumpus L.)
Source: PLoS One. 2021 Nov 22;16(11):e0259725. doi: 10.1371/journal.pone.0259725 (PMC8608339; doi:10.1371/journal.pone.0259725)
Supplement: S1 Protocol — (DOCX) [file pone.0259725.s001.docx]

**Supplementary Information**

**S1 Protocol *MALDI-TOF Main Spectra Profile (MSP) generation for P. anguilliseptica***

1µl of bacterial cells were suspended in 300µl SDW and 900µl 96% ethanol. After centrifugation for 2 min at 11357g (Eppendorf Minispin **^®^**) and removal of the supernatant, proteins were extracted from the bacterial pellet with formic acid and acetonitrile (ACN). Eight individual 1µl aliquots of each ACN extraction were then spotted onto a polished steel MALDI target plate (Bruker Daltonics), dried at room temperature, overlaid with 1µl HCCA matrix solution (saturated α-cyano-4-hyoxycinnamic acid in 50% ACN 2,5% trifluoroacetic acid) (Bruker Daltonics), and allowed to air dry. Three spectra were generated for each spot (N=24) using the MBT-AutoX method in FlexControl 3.4 (Bruker Daltonics). Peak lists were created using the MBT-Standard FAMS Method followed by baseline subtraction and smoothing in FlexAnalysis (Bruker Daltonics). Each spectrum was quality controlled for flatline spectrum, outlier peaks and peak shifts <500ppm. Spectra passing the quality check were included in the strain specific MSP generated in MBT Compass Explorer (Bruker Daltonics). MSPs were constructed based on 16-21 individual spectra per isolate. Prior to, and concurrently during, each MSP creation, the instrument was calibrated with bacterial test standard (Bruker daltonics).
