# Supplementary material for: Multilocus sequence analysis reveals different lineages of Pseudomonas anguilliseptica associated with disease in farmed lumpfish (Cyclopterus lumpus L.)
Source: PLoS One. 2021 Nov 22;16(11):e0259725. doi: 10.1371/journal.pone.0259725 (PMC8608339; doi:10.1371/journal.pone.0259725)
Supplement: S1 Table — (DOCX) [file pone.0259725.s002.docx]

Table S1: Locus accession numbers (NCBI) for P. aeruginosa, P. orysihabitans and P. anguilliseptica for alignment and PCR primer design**.**
