# Supplementary material for: Multilocus sequence analysis reveals different lineages of Pseudomonas anguilliseptica associated with disease in farmed lumpfish (Cyclopterus lumpus L.)
Source: PLoS One. 2021 Nov 22;16(11):e0259725. doi: 10.1371/journal.pone.0259725 (PMC8608339; doi:10.1371/journal.pone.0259725)
Supplement: Supplementary file 3 [file pone.0259725.s003.docx]

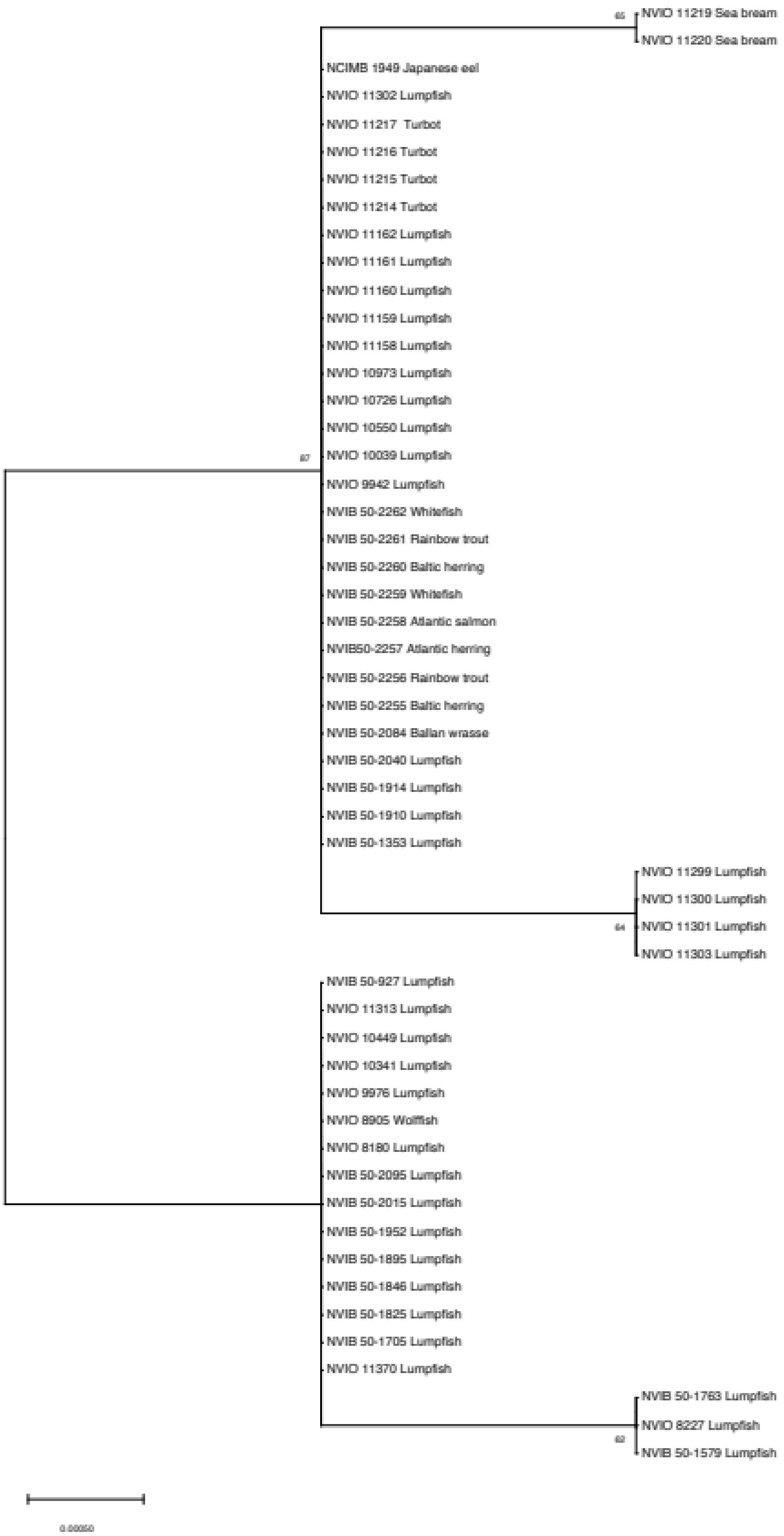

Figure S1: Maximum likelihood tree based on rpoD sequence (766 bp). Bootstrap support values shown next to the branches. Annotation: Norwegian Veterinary Institute Bergen/Oslo (NVIB/NVIO), isolate number, fish species.
