# Supplementary material for: Multilocus sequence analysis reveals different lineages of Pseudomonas anguilliseptica associated with disease in farmed lumpfish (Cyclopterus lumpus L.)
Source: PLoS One. 2021 Nov 22;16(11):e0259725. doi: 10.1371/journal.pone.0259725 (PMC8608339; doi:10.1371/journal.pone.0259725)
Supplement: Supplementary file 4 [file pone.0259725.s004.docx]

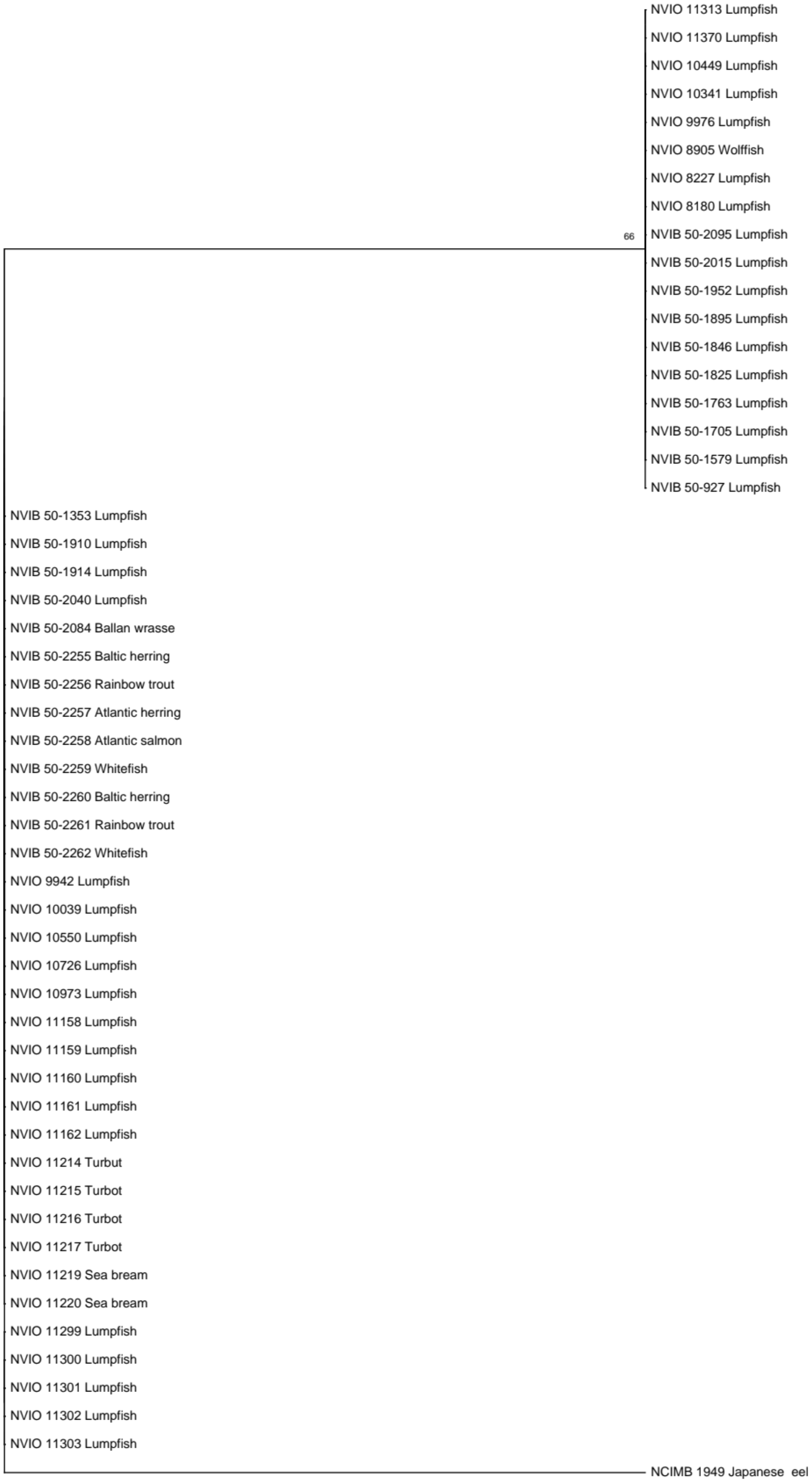
NVIO 11313 Lumpfish
NVIO 11370 Lumpfish
NVIO 10449 Lumpfish
NVIO 10341 Lumpfish
NVIO 9976 Lumpfish
NVIO 8905 Wolffish
NVIO 8227 Lumpfish
NVIO 8180 Lumpfish
66
NVIB 50-2095 Lumpfish
NVIB 50-2015 Lumpfish
NVIB 50-1952 Lumpfish
NVIB 50-1895 Lumpfish
NVIB 50-1846 Lumpfish
NVIB 50-1825 Lumpfish
NVIB 50-1763 Lumpfish
NVIB 50-1705 Lumpfish
NVIB 50-1579 Lumpfish
NVIB 50-927 Lumpfish
NVIB 50-1353 Lumpfish
NVIB 50-1910 Lumpfish
NVIB 50-1914 Lumpfish
NVIB 50-2040 Lumpfish
NVIB 50-2084 Ballan wrasse
NVIB 50-2255 Baltic herring
NVIB 50-2256 Rainbow trout
NVIB 50-2257 Atlantic herring
NVIB 50-2258 Atlantic salmon
NVIB 50-2259 Whitefish
NVIB 50-2260 Baltic herring
NVIB 50-2261 Rainbow trout
NVIB 50-2262 Whitefish
NVIO 9942 Lumpfish
NVIO 10039 Lumpfish
NVIO 10550 Lumpfish
NVIO 10726 Lumpfish
NVIO 10973 Lumpfish
NVIO 11158 Lumpfish
NVIO 11159 Lumpfish
NVIO 11160 Lumpfish
NVIO 11161 Lumpfish
NVIO 11162 Lumpfish
NVIO 11214 Turbut
NVIO 11215 Turbot
NVIO 11216 Turbot
NVIO 11217 Turbot
NVIO 11219 Sea bream
NVIO 11220 Sea bream
NVIO 11299 Lumpfish
NVIO 11300 Lumpfish
NVIO 11301 Lumpfish
NVIO 11302 Lumpfish
NVIO 11303 Lumpfish
NCIMB 1949 Japanese eel
